# Urgent vs. planned peritoneal dialysis initiation: complications and outcomes in the first year of therapy

**DOI:** 10.1590/2175-8239-JBN-2021-0182

**Published:** 2022-04-04

**Authors:** Murilo Pilatti, Valeria Catharina Theodorovitz, Daniela Hille, Gabriela Sevignani, Helen Caroline Ferreira, Marcos Alexandre Vieira, Viviane Calice-Silva, Paulo Henrique Condeixa de França

**Affiliations:** 1 Universidade da Região de Joinville, Programa de Pós-Graduação em Saúde e Meio Ambiente, Joinville, SC, Brasil.; 2 Fundação Pró-Rim, Joinville, SC, Brasil.

**Keywords:** Renal Insufficiency, Chronic, Renal Replacement Therapy, Peritoneal Dialysis, Emergencies, Insuficiência Renal Crônica, Terapia de Substituição Renal, Diálise Peritoneal, Emergências

## Abstract

**Introduction:**

Urgent-start peritoneal dialysis (US-PD) has been proposed as a safe modality of renal replacement therapy (RRT) for end-stage renal disease (ESRD) patients with an indication for emergency dialysis initiation. We aimed to compare the characteristics, 30-day complications, and clinical outcomes of US-PD and planned peritoneal dialysis (Plan-PD) patients over the first year of therapy.

**Methods:**

This was a single-center retrospective study that included incident adult patients followed for up to one year. US-PD was considered when incident patients started therapy within 7 days after Tenckhoff catheter implantation. Plan-PD group consisted of patients who started therapy after the breaking period (15 days). Mechanical and infectious complications were compared 30 days from PD initiation. Hospitalization and technique failure during the first 12 months on PD were assessed by Kaplan-Meier curves and the determinants were calculated by Cox regression models.

**Results:**

All patients starting PD between October/2016 and November/2019 who fulfilled the inclusion criteria were analyzed. We evaluated 137 patients (70 in the US-PD x 67 Plan-PD). The main complications in the first 30 days were catheter tip migration (7.5% Plan-PD x 4.3% US-PD - p= 0.49) and leakage (4.5% Plan-PD x 5.7% US-PD - p=0.74). Most catheters were placed using the Seldinger technique. The main cause of dropout was death in US-PD patients (15.7%) and transfer to HD in Plan-PD patients (13.4%). The occurrence of complications in the first 30 days was the only risk factor for dropout (OR = 2.9; 95% CI 1.1-7.5, p = 0.03). Hospitalization rates and technique survival were similar in both groups.

**Conclusion:**

The lack of significant differences in patients’ outcomes between groups reinforces that PD is a safe and applicable dialysis method in patients who need immediate dialysis.

## Resumo

**Introdução:**

A diálise peritoneal de início urgente (US-PD) foi proposta como modalidade segura de terapia renal substitutiva (TRS) para pacientes com doença renal em estágio 5 (DRC-5) com indicação de início de diálise de emergência. Buscamos comparar características, complicações em 30 dias e desfechos clínicos de pacientes em US-PD e diálise peritoneal planejada (DP-plan) no primeiro ano de terapia.

**Métodos:**

Estudo retrospectivo de centro único, que incluiu pacientes adultos incidentes em DP acompanhados por até um ano. Considerou-se US-PD quando os pacientes iniciaram terapia até 7 dias após implante do cateter Tenckhoff. O grupo DP-plan consistiu de pacientes iniciando terapia após período break-in (15 dias). Compararam-se complicações mecânicas e infecciosas 30 dias após o início da DP. Hospitalização e falha da técnica durante os primeiros 12 meses em terapia foram avaliados por curvas Kaplan-Meier e os seus determinantes foram analisados por modelos de regressão de Cox.

**Resultados:**

Analisaram-se todos os pacientes iniciando DP entre Outubro/2016-Novembro/2019 que preencheram os critérios de inclusão. Avaliamos 137 pacientes (70 US-PD x 67 DP-plan). As principais complicações nos primeiros 30 dias foram migração da ponta do cateter (7,5% DP-plan x 4,3% US-PD - p= 0,49) e extravasamento (4,5% DP-plan x 5,7% US-PD - p=0,74). A maioria dos cateteres foi implantada pela técnica de Seldinger. A principal causa de saída da terapia foi óbito em pacientes em US-PD (15,7%) e transferência para HD em pacientes em DP-plan (13,4%). A ocorrência de complicações nos primeiros 30 dias foi o único fator de risco para saída da terapia (OR = 2,9; IC 95% 1,1-7,5, p = 0,03). Taxas de hospitalização e sobrevida da técnica foram similares em ambos os grupos.

**Conclusão:**

A ausência de diferenças significativas nos desfechos dos pacientes entre os grupos reforça que DP é um método de diálise seguro e aplicável em pacientes que necessitam diálise imediata.

## Introduction

Peritoneal dialysis (PD) has been used for patients with stage 5 chronic kidney disease (CKD-5) as renal replacement therapy (RRT) for more than 4 decades^1^. PD is a home-based therapy that brings quality of life and autonomy to patients. It is also considered an effective and less expensive alternative to guarantee access to RRT^2^ and is the modality of choice for patients who cannot obtain vascular access and tolerate hemodialysis (HD)^3,4^.

These factors, associated with well-documented satisfactory outcomes, make PD an interesting RRT modality worldwide, especially in areas with poor access to pre-dialysis care, where there is a lack of screening and monitoring of individuals at higher risk to develop CKD and rapid residual renal function deterioration may happen in some patients who require urgent dialysis initiation^5^. PD offers many advantages, such as eliminating the need for a central venous catheter (CVC) therefore preserving vascular access, reducing intradialytic hemodynamic effects on patients, helping to preserve residual renal function for a longer time, and others.

Although the data available on urgent-start peritoneal dialysis (US-PD) are relatively recent, they indicate that mortality is at least similar to that of patients treated with unplanned HD^6^. In addition, complications and outcomes of US-PD are equivalent to those of patients undergoing planned peritoneal dialysis (Plan-PD), indicating the safety of using US-PD in the treatment of chronic patients who require urgent dialysis initiation^7-10^. Considering the lack of HD centers in most countries, the use of US-PD would also allow nephrologists to treat a larger number of patients and shorten the waiting list for HD places^7,11^.

With this in mind, we aimed to compare patients undergoing US-PD and Plan-PD regarding their demographic and clinical characteristics, 30-day therapy complications, and complications and outcomes during one-year follow-up considering hospitalization and therapy dropout.

## Methods

### Study site

This was a retrospective cohort study carried out in a single-center PD outpatient clinic in Joinville, Santa Catarina, Brazil. This dialysis unit treats about 400 ESRD patients, the majority of whom (75%) are on hemodialysis. There is no waiting list for dialysis, and patients start either HD or PD right after the referral to the facility. For this study, adult ESRD patients followed at this PD service and incidents on PD between October 1, 2016, and November 30, 2019, were included. For data collection, patients’ charts were reviewed and the information needed to answer the study research questions were assessed and analyzed.

### Groups definition

US-PD group consisted of patients that had an indication for urgent dialysis initiation, started PD within 7 days after Tenckhoff catheter implantation, and did not receive HD prior to PD. Plan-PD group consisted of patients prepared for RRT-PD who started therapy in a planned matter after 15 days of catheter implantation. Patients who migrated to PD after previous use of emergency HD were excluded from these analyses to avoid potential interference of that period on patients’ outcomes.

### Collected variables

Sociodemographic (age, sex, self-reported race, education level) and clinical (comorbidities and PD-related information) data of all participants were collected from medical records. Complications within first 30 days of PD initiation, later complications, technique failure, and hospitalization during the first year on PD were also evaluated. Early mechanical complications included leakage, bleeding, visceral perforation, and catheter tip migration. Peritonitis and exit-site infection were considered infectious complications. Regarding mechanical complications after 30 days on therapy, only information on catheter tip migration was collected, once other mechanical complications are not common after that period.

### Statistical analyses

Descriptive data were reported as mean ± standard deviation or median and IQR and as a percentage according to each variable characteristics and distribution. To compare the sociodemographic and clinical characteristics between the Plan-PD and US-PD groups, ANOVA or independent sample t-tests were used, according to the number of quantitative categories, and chi-square test was used for categorical variables. Kaplan-Meier curves were constructed to assess hospital-free survival and PD survival over the first year of follow-up in both groups, and comparisons were conducted using Log-rank test. Cox regression adjusted for confounding variables such as age, sex, self-reported race and education, comorbidities, and catheter implantation technique. Hospitalization due to early and late complications (mechanical and infectious ones) during the first year follow-up and first fill volume was used to assess variables associated with outcomes in both groups separately. The variables included in the model were chosen based on their clinical relevance to the study outcomes. Statistical analyses were performed using SPSS software (IBM) version 26. A p-value <0.05 was considered statistically significant.

### Ethics

The study was submitted to the Research Ethics Committee (CEP) of UNIVILLE and approved according to the guidelines in Resolution 466/2012 of the National Health Council (Opinion 3.089.933). The study was also approved by the committees of the co-participating institution.

## Results

**Figure 1:**
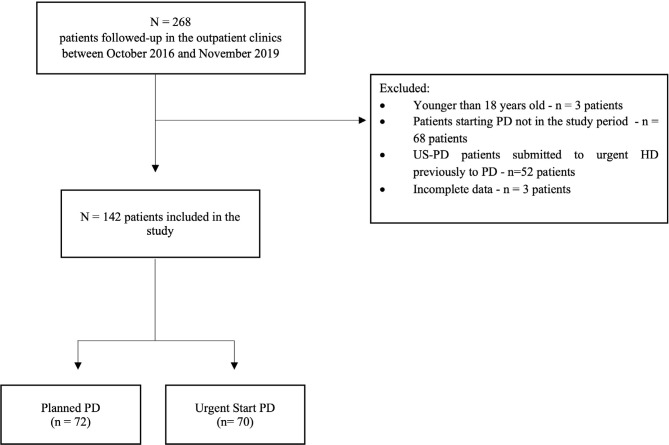
Study flowchart.

Of the 268 patients followed-up at the PD center in the above period, 137 were included in the study, 70 (51.1%) in the US-PD group and 67 (48.9%) in the Plan-PD group, who were followed up for a median of 9.4 months, with the shortest follow-up being 31 days and the longest 38 months. The study flowchart is shown in Figure 1.

US-PD: Urgent start peritoneal dialysis, HD: hemodialysis.

### Clinical and sociodemographic characteristics

The mean age was 54 ± 15 years, and age ranged from 20 and 87 years. Patients from the Plan-PD group were older than those from the US-PD group. There was a balance in gender distribution among patients (55% male and 45% female), with no significant difference between groups. Arterial hypertension (HTN) and diabetes mellitus (DM) were the most prevalent diseases, affecting 83.2 and 42.3% of patients, respectively, distributed similarly between groups. Table 1 shows these and other clinical and sociodemographic characteristics of the study patients.

**Table 1:** Sociodemographic and clinical characteristics of the studied PD patients

Variable	All patients (n=137)	US-PD (n=70)	Plan-PD (n=67)	p
**Age in years (mean ± SD)**	54 (±15)	51.7 (±14.7)	56.4 (± 15)	0.06
**Male, n (%)**	76 (55.5)	36 (51.4)	40 (59.7)	0.33
**Skin color - white, n (%)**	128 (93.4)	65 (92.9)	63 (94)	0.79
**Education, n (%)**				0.21
Elementary school	32 (25.8)	14 (20.9)	18 (31.6)	
Middle school	39 (31.5)	21 (31.3)	18 (31.6)	
High school	39 (31.5)	26 (38.8)	13 (22.8)	
University or higher	14 (11.3)	6 (9)	8 (14)	
**Hypertension, n (%)**	114 (83.2)	58 (84.1)	56 (84.8)	0.89
**Diabetes, n (%)**	58 (42.3)	26 (44.2)	32 (48.5)	0.21
**Follow-up in months (median, IQR)**	9.4 (3.9-18.7)	8.7 (3.8-16.8)	11.5(4.4-20.1)	0.49

US-PD: urgent start peritoneal dialysis, Plan-PD: planned peritoneal dialysis, SD: standard deviation, IQR: interquartile range.

### Technical aspects of implementing peritoneal dialysis

The technical aspects related to the catheter implantation procedure are shown in Table 1 - supplementary material. Approximately 50% of the patients undergoing Plan-PD migrated from hemodialysis to PD. The technique for catheter implantation was based on the patient’s abdomen characteristics and previous surgical history, being either the Seldinger, mini-laparotomy, or videolaparoscopy technique. The latter was performed only by trained surgeons. Purse string suture is not done routinely in catheter implantation. There was a slight predominance of the use of the Seldinger technique for implantation of the Tenckhoff catheter, especially in cases requiring urgent dialysis initiation. The initial dialysis fill volume was similar for both groups (Table 1- Supplementary material).

### Complications related to peritoneal dialysis

There were no infectious complications in the first 30 days of PD. Non-infectious complications occurred in 7 (10%) patients from the US-PD group and 10 (13.8%) patients from the Plan-PD group, including 2 patients who had immediate complications after catheter implantation (one case of bleeding and another of bowel perforation). These patients were promptly submitted to emergency surgery with immediate damage control and maintenance of PD as dialysis therapy. The main mechanical complications in the first 30 days were catheter tip migration (7.5% in Plan-PD vs. 4.3% in US-PD - p=0.49) and leakage (4.5% in Plan-PD vs. 5.7% in US-PD- p=0.74). After the 30th day on PD, 30 (22%) patients in both groups had some catheter-related infectious complications (peritonitis or exit-site infection). All patients diagnosed with peritonitis started treatment in a hospital setting. The complications observed before and after the 30th day of PD are shown in Table 2.

**Table 2:** Mechanical and infectious complications related to peritoneal dialysis

Complications	All (n=137)	US-PD (n=70)	Plan-PD (n=67)	p
**First 30 days on PD, n (%)**	15 (10.9)	6 (8.6)	9 (13.4)	0.36
Catheter tip migration	8 (5.6)	3 (4.3)	5 (7.5)	0.49
Leakage	7 (5)	4 (5.7)	3 (4.5)	0.74
Bleeding	1 (0.7)	0 (0)	1 (0.7)	0.49
Visceral perforation	1 (0.7)	0 (0)	1 (0.7)	0.49
Peritonitis	0 (0)	0 (0)	0 (0)	
Catheter exit-site infection	0 (0)	0 (0)	1 (0)	0.49
**After 30 days on PD, n (%)**	49 (35.7)	26 (37,1)	23 (34.3)	0.33
Catheter tip migration	19 (13.8)	12 (17.1)	7 (10.4)	0.21
Peritonitis	15 (10.9)	9 (12.8)	6 (8.9)	0.45
Catheter exit-site infection	15 (10.9)	5 (7.1)	10 (14.9)	0.14

US-PD: diálise peritoneal de início urgente, DP-plan: diálise peritoneal planejada.

### Hospitalization and technique survival

Approximately 22% of studied PD patients were hospitalized at least once during the 12-month follow-up period and 33 (24%) patients experienced complications that determined technique dropout. Hospital-free survival in the first year of PD was 77.1% in the US-PD group and 78.8% in the Plan-PD group.

The main reason for dropout in the US-PD group was death in 11 (15.7%) patients and transfer to HD in 9 (13.4%) patients in the Plan-PD group. Twenty-five (18.2%) patients withdrew from the assigned dialysis method for positive reasons such as kidney transplantation and recovery of residual renal function. The technique survival rates found for the US-PD and Plan-PD groups were 75.7% and 77.3%, respectively, in the first year.

**Figure 2:**
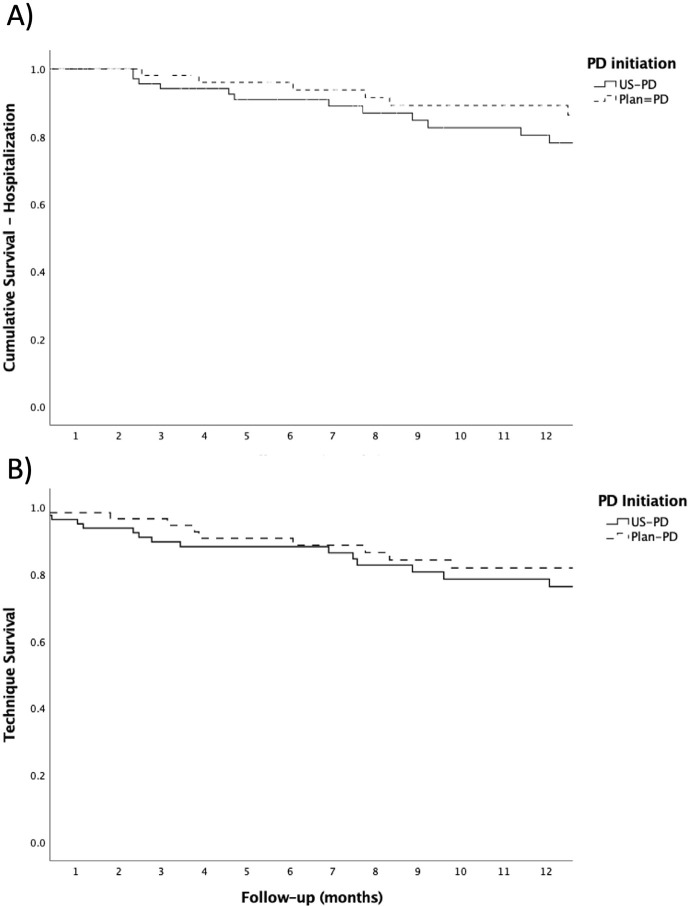
Kaplan-Meier curve showing a) all-cause hospitalization and b) technique survival during the first year on PD in US-PD and Plan-PD.

The reasons for leaving the assigned method are shown in Table 3. Kaplan-Meier curves in Figures 2a and 2b graphically demonstrate the cumulative hospitalization-free survival and technique survival in the first year of follow-up.

US-PD: urgent start peritoneal dialysis, Plan-PD: planned peritoneal dialysis

**Table 3:** Hospitalization and dropout during the first year on therapy

Outcomes	All (n=137)	US-PD (n=70)	Plan=PD (n=67)	p
**Hospitalizations, n (%)**	30 (21.9)	16 (22.9)	14 (20.9)	0.78
**Dropout, n (%)**				
**Negative cause**	33 (24.1)	18 (25.7)	15 (22.3)	0.68
Death	17 (12.4)	11 (15.7)	6 (9.0)	0.23
Transfer to HD	16 (11.7)	7 (10)	9 (13.4)	0.53
**Positive cause, n (%)**	25 (18.2)	15 (21.4)	10 (14.5)	0.32
Kidney transplantation	22 (16.1)	13 (18.6)	9 (13.4)	0.41
Recovery of kidney function	3 (2.2)	2 (2.9)	1 (1.5)	0.58
Migração da ponta do cateter	19 (13,8)	12 (17,1)	7 (10,4)	0,21
Peritonite	15 (10,9)	9 (12,8)	6 (8,9)	0,45
Infecção no sítio de saída do cateter	15 (10,9)	5 (7,1)	10 (14,9)	0,14

US-PD: urgent start peritoneal dialysis, Plan-PD: planned peritoneal dialysis, HD: hemodialysis.

Cox’s regression analyses were performed to identify risk factors for dropout and hospitalization in both groups. The occurrence of complications in the first 30 days was identified as a risk factor in the US-PD group, with a relative risk of 2.9 (95% CI 1.1-7.5; p = 0.03). In the Plan-PD- group, catheter implantation by laparotomy technique (OR 4.5; 95% CI 1.0-21; p = 0.05) were identified as a risk factor for PD dropout. No risk factors for all-cause hospitalization were identified during the follow-up period in both groups.

## Discussion

Our findings demonstrate that there were no sociodemographic or clinical differences between the US-PD and Plan-PD groups. More interestingly, there were no significant differences in 30-day complications, hospitalizations, and technique survival during the first year on therapy for patients who started urgent PD compared to Plan-PD initiation, indicating the efficiency and safety of PD in urgent situations, which is similar to most studies carried out on the topic^5,7,10,12-14^.

The most frequent early complications found in the studied groups were catheter tip migration and leakage through the catheter exit site. There was no significant difference between the US-PD and Plan-PD groups in this regard. Our early complication findings are comparable to those published in the international literature^8, 15-17^ and in Brazil^13, 17-21^. In our study, the occurrence of complications in the first 30 days was a significant risk factor for dropout in the first year in the US-PD group, with a relative risk of 2.8 (95% CI 1.12-7.03; p=0.03).

There were no infectious complications before the 30th day of our study, corroborating the results found in the main systematic reviews and meta-analyzes published recently. Early infectious events were considered rare, occurring in 0 to 2.5% of cases^10, 22^. Also, about 22% of patients had infectious complications at some point after 30 days on PD. Fifteen of them (11%) had peritonitis and another 15 (11%) had an exit-site infection or tunnel infection during one year of follow-up. About 9% of the patients on Plan-PD had peritonitis, while approximately 13% of those allocated to US-PD had peritonitis. The total peritonitis rate during the first year on therapy was 0.110 episodes/patient-year and was not different between groups (0.128 episodes/patient-year in US-PD and 0.090 episodes/patient-year in Plan-PD; p = 0.45). This incidence is below the recommendation by the International Society of Peritoneal Dialysis (ISDP)^23^.

The occurrence of exit site or tunnel infection was similar in the two groups and close to 11%.; such findings are in line with what is presented in the literature^8,24,25^.

About 22% of patients were hospitalized in the first year of PD, with no significant difference between the US-PD and Plan-PD groups (22.9% and 20.9%, respectively; p = 0.8) which is comparable to the available literature^26^. Technique survival in the first year on PD was 75.7% in the US-PD group and 77.3% in the Plan-PD group, which is slightly below the 80% recommended by ISPD^27^. Our result is similar to other Brazilian studies that report a technique survival of around 86% in the first 90 and 180 days of PD and that considered the same period for PD initiation as US-PD (up to 7 days after catheter implantation)^18,24^.

The main dropout reason was death, as 12% of PD patients died (15.7% US-PD vs. 9% Plan-PD; p=0.3), similar to what is observed in the literature (25 to 34%),^8, 22, 28^. The occurrence of complications in the first 30 days was the only risk factor for technique dropout in the US-PD group. Catheter implantation by laparotomy was a risk factor for technique dropout in the Plan-PD group, which may be related to the complexity of the patient’s abdomen that poses a greater risk for catheter malfunction and technique failure^27, 29, 30^. In our study, 16% of patients on PD were submitted to kidney transplantation along the first year on PD, which is also similar to previous studies that show that 20 to 26% of patients receive a transplant^8,28^.

Our study had some limitations, such as being a non-randomized single-center study with a small sample size, which affects the generalizability of our findings. In addition, the patients’ clinical circumstances at the time of dialysis initiation could not be recovered from the data charts, making comparisons between groups difficult. However, the study had some strengths, such as the definition of urgent-start PD of up to 7 days of catheter implantation rather than up to 14 days as in most studies in the literature. This may allow better characterization of early complications. In addition, the one-year follow-up period allowed us to evaluate data on later outcomes such as hospitalization, technique failure, and infectious complications, whereas most articles published on this topic follow up patients for a shorter period.

## Conclusion

Demographic and clinical characteristics, 30-day complications, and first-year outcomes were similar in patients starting urgent PD compared to those starting planned PD. These findings corroborate the literature, showing that PD is a safe and applicable dialysis method in patients who need urgent dialysis.

## Supplementary Material

The following online material is available for this article:

Table 1 - Catheter implantation and PD initiation.
