# Supplementary material for: Urgent vs. planned peritoneal dialysis initiation: complications and outcomes in the first year of therapy
Source: J Bras Nefrol. 2022 Apr 4;44(4):482–9. doi: 10.1590/2175-8239-JBN-2021-0182 (PMC9838670; doi:10.1590/2175-8239-JBN-2021-0182)
Supplement: Supplementary file 1 [file 2175-8239-jbn-2021-0182-suppl.pdf]

Braz. J. Nephrol. (J. Bras. Nefrol.)
https://doi.org/10.1590/2175-8239-JBN-2021-0182

Supplementary Material to "Urgent vs. planned peritoneal dialysis initiation: complications and outcomes in the first year of therapy"

Table 1 – Catheter implantation and PD initiation.

Variable	All (n=137)	US-PD (n=70)	Plan-PD (n=67)	p
**Previous HD, n (%)**	33 (24.1)	0 (0)	33 (49.2)	<0.001
**Implantation technique, n %**				0.27
Seldinger	77 (56.2)	44 (62.9)	33 (49.3)	
Laparotomy	42 (30.7)	18 (25.7)	24 (35.8)	
Laparoscopy	18 (13.1)	8 (11,4)	10 (14.9)	
**First-fill volume, mL (mean ± SD)**	1,868 (±206)	1,874 (±168)	1,854 (±244)	0.59

US-PD: urgent-start peritoneal dialysis, Plan-PD: planned peritoneal dialysis, HD: hemodialysis, SD: standard deviation.
